# A case of peripancreatic plexiform schwannoma

**DOI:** 10.1186/s40792-021-01284-7

**Published:** 2021-08-28

**Authors:** Kenji Fukushima, Makoto Shinzeki, Kentaro Tai, Masaki Omori, Natsuko Yamauchi, Tomoko Tanaka, Yasunori Matsuda, Hiroshi Ashitani, Kenichi Tanaka

**Affiliations:** Department of Surgery, Osaka Saiseikai Nakatsu Hospital, 2-10-39, Shibata, Kita-ku, Osaka, 530-0012 Japan

**Keywords:** Plexiform schwannoma, Peripancreatic plexus, Splenic plexus, Laparoscopic pancreatectomy, Pancreas, Neurilemmoma

## Abstract

**Background:**

Plexiform schwannoma is one of the least common variants of schwannoma, accounting for only 5% of all schwannoma cases. It generally occurs in the skin and subcutaneous tissues and is uncommon in deep soft tissue or viscera. We present an extremely rare case of plexiform schwannoma arising from the peripancreatic plexus.

**Case presentation:**

A 29-year-old man presented with hyperglycemia detected during a medical checkup. He was diagnosed with type 1 diabetes based on the clinical findings and laboratory tests. During the diagnostic process for diabetes, a 2.5 cm mass was incidentally detected in the pancreas by abdominal ultrasound. Contrast-enhanced computed tomography revealed a mass that was gradually enhanced at the body and tail of the pancreas. Magnetic resonance imaging revealed low signal intensity of the mass on T1-weighted images and high signal intensity on T2-weighted and diffusion-weighted images. Magnetic resonance cholangiopancreatography showed no abnormal findings in the main pancreatic duct. Endoscopic ultrasonography (EUS) showed a lobulated, low-echoic mass with a clear boundary. EUS-guided fine needle biopsy was performed, and spindle-shaped cells that were diffusely immunopositive for S-100 and negative for c-kit and desmin were detected, resulting in a diagnosis of a neurogenic tumor arising from the pancreas or the peripancreatic nerve plexus. The patient underwent laparoscopic spleen-preserving distal pancreatectomy. Although the tumor was connected to the splenic plexus, the splenic artery could be divided along its adventitial plane. Macroscopic findings of the excised tumor consisted of multiple yellowish-white nodules, and its histopathological features were consistent with plexiform schwannoma. There was no pancreatic tissue on the dorsal surface of the tumor, which suggested that the tumor arose from the peripancreatic nerve plexus.

**Conclusions:**

The findings documented herein can aid in the differential diagnosis of peripancreatic schwannoma and in planning appropriate treatment.

## Background

Schwannomas are benign, encapsulated neurogenic tumors originating from the Schwann cells of the peripheral nerve sheath. There are several morphological subtypes of schwannoma, such as conventional, cellular, ancient, plexiform, melanotic, and microcystic/reticular subtypes [1, 2]. Plexiform schwannoma is one of the least common variants of schwannoma and is characterized by a multinodular (plexiform) growth pattern that accounts for up to 5% of all schwannomas [2, 3].

Most cases of plexiform schwannoma generally occur in the skin and subcutaneous tissues of the head, neck arms, and chest and are uncommon in the deep soft tissue or viscera [2, 3]. To our knowledge, peripancreatic localization has not been reported in the English literature. We present the first known case of plexiform schwannoma arising from the peripancreatic plexus.

## Case presentation

A 29-year-old man presented with hyperglycemia detected during a medical checkup. He was diagnosed with type 1 diabetes based on the clinical findings and laboratory tests. During the diagnostic process for diabetes, a 2.5-cm mass was incidentally detected at the body and tail of the pancreas by abdominal ultrasound. He had no symptoms or dermal lesions, and his abdomen was soft with no evidence of a palpable mass. There was no past or family history of neurofibromatoses. Tumor markers (carcinoembryonic antigen and carbohydrate antigen 19–9) were within normal ranges.

**Fig. 1:**
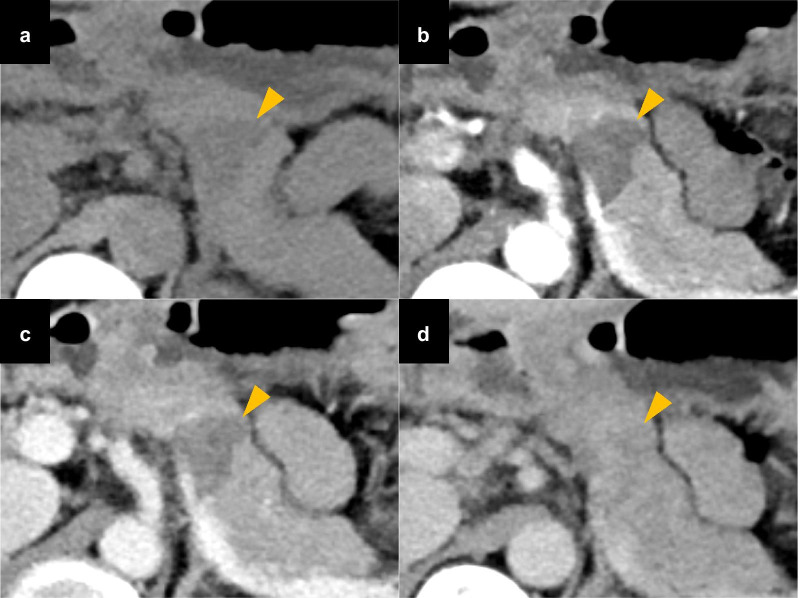
Contrast-enhanced computed tomography showing a gradually enhanced tumor (triangles) at the pancreatic body and tail. **a** Plain. **b** Arterial phase. **c** Portal phase. **d** Delayed phase

**Fig. 2:**
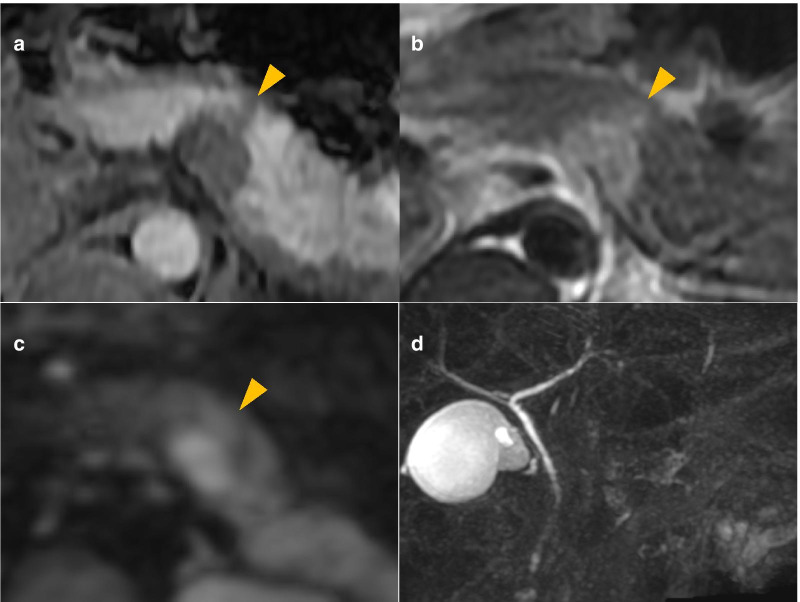
Magnetic resonance imaging revealed a mass (triangles) exhibiting hypointensity on T1-weighted images (**a**), hyperintensity on T2-weighted images **b** and diffusion-weighted images **c** at the pancreas body and tail. Magnetic resonance cholangiopancreatography showed no dilation of the pancreatic duct (**d**)

**Fig. 3:**
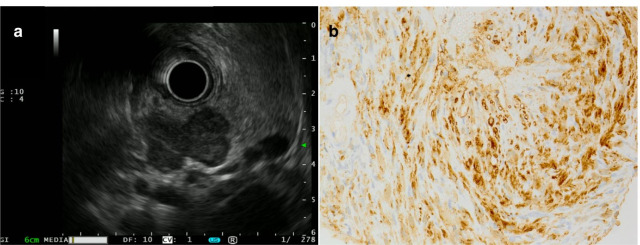
Endoscopic ultrasonography (EUS) showed a lobulated, low-echoic mass with a clear boundary at the pancreatic body and tail (**a**). The tumor cells in the EUS-fine needle biopsy specimens were spindle shaped and diffusely immunopositive for S-100 (**b**)

Contrast-enhanced computed tomography (CT) revealed a mass at the body and tail of the pancreas. The mass was gradually enhanced, and the tumor border was clearly demonstrated (Fig. 1). Magnetic resonance imaging (MRI) revealed low signal intensity of the mass on T1-weighted images and high signal intensity on T2-weighted and diffusion-weighted images (Fig. 2a–c). Magnetic resonance cholangiopancreatography showed no abnormal findings in the main pancreatic duct (Fig. 2d). Endoscopic ultrasonography (EUS) showed a lobulated, low-echoic mass with a clear boundary (Fig. 3a). EUS-guided fine needle biopsy was performed, and spindle-shaped cells that were strongly immunopositive for S-100 (Fig. 3b) and negative for c-kit and desmin were detected. The Ki-67 positivity rate was less than 5%. According to these findings, we diagnosed the mass as a neurogenic tumor, particularly schwannoma arising from intra- or extrapancreatic nerve fibers.

**Fig. 4:**
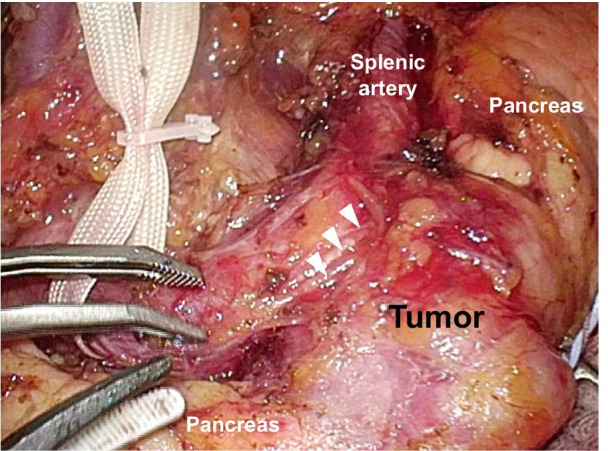
Intraoperative photograph after taping the splenic artery. The tumor was observed at the body and tail of the pancreas and was tightly connected to the splenic plexus (triangles)

It was unclear whether the location of the tumor was inside or outside the pancreas, and it seemed difficult to divide the tumor from the parenchyma of the pancreas, so we planned a laparoscopic distal pancreatectomy instead of tumor excision. Intraoperatively, the tumor was observed at the body and tail of the pancreas and was tightly connected to the splenic plexus (Fig. 4). The tumor could be easily dissected from the adventitia of the splenic artery. Therefore, we ultimately performed laparoscopic spleen-preserving distal pancreatectomy with conservation of the splenic artery and vein.

**Fig. 5:**
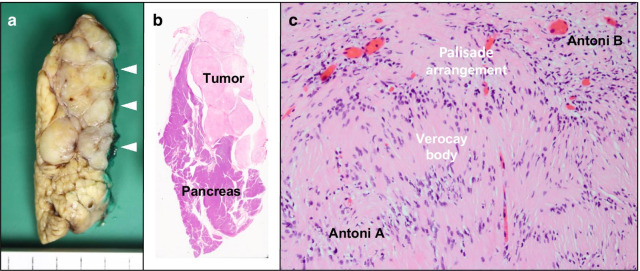
Macroscopic and microscopic findings of the resected specimen. **a** Cut surface of the resected specimen showed that the tumor was white-yellowish, well-demarcated, and multinodular. There was no pancreatic parenchyma on the dorsal surface of the tumor (triangles). **b** Loupe image. **c** The tumor cells were spindle-shaped and had a palisading arrangement, forming structures called Verocay bodies in hypercellular Antoni A areas. Hypocellular areas, called Antoni B areas, were also visible. Mitosis was rarely seen. Hematoxylin–eosin staining, original magnification 200 ×

Macroscopically, the excised tumor was 3.2 × 1.5 cm in size and consisted of multiple yellowish-white nodules surrounded by a thin fibrous capsule (Fig. 5a, b). Microscopically, the lesion mainly consisted of hypercellular areas composed of fascicular and interlacing proliferation of spindle cells with indistinct cytoplasmic borders (Antoni A areas). In these areas, the cell nuclei were aligned in palisades with the formation of Verocay bodies. Hypocellular areas with loose stroma (Antoni B areas) were also occasionally observed, while mitosis was rarely seen (Fig. 5c). These findings confirmed a diagnosis of plexiform schwannoma. There was no pancreatic parenchyma on the dorsal surface of the tumor (Fig. 5a, b), which suggested that the tumor arose from the peripancreatic nerve plexus.

After surgery, the patient recovered uneventfully and was discharged on postoperative day 10. The patient was followed for 34 months and continued to do well without any signs of recurrence or complications at the last follow-up.

## Discussion

Plexiform schwannoma is a rare benign peripheral nerve sheath tumor first described in 1978 by Harkin et al. that macroscopically grows in a multinodular or plexiform pattern [4, 5]. Plexiform schwannoma usually develops in the skin or superficial soft tissue and rarely in deep-seated nerves [2, 3]. Cases of this tumor originating in uncommon sites, such as the deep soft tissue and gastrointestinal tract, have been documented but are scarce [6–9]. We herein report the first case of plexiform schwannoma originating from the peripancreatic nerve plexus.

According to a review of plexiform schwannoma by Iida et al., the age of the patients can range from 2 to 80 years (mean, 30 years), with no pronounced gender predominance [8]. Most plexiform schwannomas present as single, soft to rubbery, movable, nontender, and sometimes painful nodules less than 2.5 cm in diameter [10]. Neurofibromatoses, including neurofibromatosis type 1, neurofibromatosis type 2, and schwannomatosis, are genetic neurogenetic disorders characterized by the development of multiple nerve sheath tumors [11]. Plexiform schwannoma mostly occurs sporadically, as in our case, occasionally in patients with neurofibromatosis type 2 or schwannomatosis and rarely in patients with neurofibromatosis type 1 [3]. Our patient had a solitary tumor and no clinical findings or familial history of neurofibromatoses.

Histologically, most plexiform schwannomas have the essential features of conventional schwannoma except for a multinodular growth pattern. These include composition solely of spindle-shaped Schwann cells, a fibrous capsule, hyaline vessels, cellular (Antoni A) and loose-textured (Antoni B) areas, and Verocay bodies (opposing rows of spindle nuclei separated by anucleate rows of eosinophilic processes). In plexiform schwannoma, Antoni A areas generally predominate, while Antoni B areas are less frequent. Degenerative changes such as necrosis, cyst formation, and hemorrhage are uncommon [2, 6, 12]. Similar to all schwannomas, the tumor cells of plexiform schwannoma are uniformly positive for S-100 on immunohistochemistry, while they are negative for c-kit, smooth muscle actin, and desmin [6, 8]. These features are consistent with the findings in our case.

Plexiform schwannoma shows complex multinodular growth and often involves multiple nerve fascicles, which differs from the single nerve fascicle involvement of conventional schwannoma [13, 14]. Although plexiform schwannoma also shows a chronic course that reflects its histopathologically benign nature as well as conventional schwannoma, plexiform schwannomas arising from major peripheral nerves often cause motor deficits with poor functional prognosis due to the difficulty of their total resection [2, 14]. Preoperative magnetic resonance imaging is useful for distinguishing between plexiform and conventional schwannomas. Plexiform schwannoma has the morphological features suggestive of multinodular configuration in contrast to conventional schwannoma with generally globular shape. In addition, conventional schwannoma demonstrates marked enhancement after gadolinium administration, while plexiform schwannoma shows somewhat less predictable enhancement [14].

It is crucial to differentiate plexiform schwannoma from plexiform neurofibroma because the latter carries a risk of malignant transformation (2–5%), in contrast to the former [15, 16]. The multinodular growth pattern of plexiform schwannoma can mimic that of plexiform neurofibroma, although plexiform neurofibroma is essentially pathognomonic for neurofibromatosis type 1 and usually occurs in early childhood. Since plexiform schwannoma and plexiform neurofibroma exhibit similar findings on CT and MRI, the final distinction between them depends on the histopathological examination [17, 18]. S-100 immunostaining is helpful since plexiform schwannoma shows diffuse and strong positivity with S-100. On the other hand, plexiform neurofibroma lacks Antoni A and B areas of schwannoma and shows weak, patchy S-100 positivity [19].

Plexiform schwannoma is a benign, noninfiltrating tumor, so the prognosis is favorable when complete excision is achieved, although recurrence has been reported in cases of incomplete resection [2, 20]. Kuo et al. reported 17 cases of peripancreatic schwannoma, although the subtypes of schwannoma were not mentioned [21]. In that report, 14 patients were treated by tumor excision with no recurrence, while concomitant pancreatectomy was performed in only 3 patients. In our case, tumor excision without pancreatectomy was considered to be a sufficient treatment as a result. However, concomitant pancreatectomy seems to be unavoidable when it is difficult to distinguish peripancreatic schwannoma from pancreatic tumors pre- or intraoperatively, as in our patient and other case reports [22, 23].

The managements of peripancreatic and pancreatic schwannomas are guided by not only anatomical locations, but also histological results. Accurate preoperative diagnoses of peripancreatic and pancreatic schwannoma are difficult due to the nonspecific and variable radiographic appearances of schwannomas, even with the use of multiple imaging modalities. They may mimic other, more common pancreatic lesions, such as pancreatic neuroendocrine tumors, mucinous cystic neoplasms, solid pseudopapillary neoplasms, mucinous cystadenocarcinomas, serous cystic neoplasms, acinar cell carcinomas, and pancreatic pseudocysts [24]. EUS-guided fine needle biopsy is useful to establish precise preoperative diagnosis and avoid unnecessary extensive radical resection [25, 26], although its high false-negative rate and the difficulty of performing biopsy for cystic or small lesions are problems [24, 27, 28]. In our case, EUS-guided fine needle biopsy provided an accurate diagnosis, resulting in a clinically reasonable treatment.

## Conclusions

In conclusion, we have described an extremely rare case of plexiform schwannoma arising from the peripancreatic plexus. The findings documented herein will aid in the differential diagnosis of peripancreatic schwannoma and in planning appropriate treatment.

## Data Availability

Not applicable.

## Abbreviations

EUS: Endoscopic ultrasonography
CT: Computed tomography
MRI: Magnetic resonance imaging
